# Understanding the Role of PBAT Content and Raster Orientation on the Mechanical Performance of Material Extrusion 3D-Printed PLA/PBAT Objects

**DOI:** 10.3390/polym18030339

**Published:** 2026-01-27

**Authors:** Sándor Kálmán Jakab, András Lajos Nagy, László Lendvai

**Affiliations:** 1 Department of Materials Engineering and Machine Design, Széchenyi István University, H-9026 Győr, Hungary; jakab.sandor.kalman@sze.hu; 2 Sustainable Polymer Engineering Research Group, Széchenyi István University, H-9026 Győr, Hungary; 3 Department of Vehicle Propulsion Technology and Power Electronics, Széchenyi István University, H-9026 Győr, Hungary; nagy.andras1@sze.hu

**Keywords:** poly(lactic acid), poly(butylene adipate-co-terephtalate), material extrusion, fused deposition modeling, fused filament fabrication, 3D printing, raster angle, mechanical properties

## Abstract

Poly(lactic acid) (PLA) is the most widely used feedstock in material extrusion (MEX) 3D printing. In this study, PLA was combined with 0–40 wt.% of poly(butylene adipate-co-terephtalate) (PBAT) to improve its ductility. The resulting blends were processed into filaments suitable for MEX 3D printing and used to fabricate specimens for mechanical characterization using three distinct raster angles (RAs; 0°, ±45°, and 90°) to statistically evaluate the individual and joint effects of blend composition and raster orientation. Melt flow index (MFI) measurements showed that increasing PBAT content reduced the MFI from 40.4 g/10 min to 34.4 g/10 min, which led to weaker bonding between printed beads, as shown in scanning electron microscopic images. Tensile strength, modulus, and impact strength were evaluated using tensile and Charpy tests. Statistical analysis showed that RA, PBAT concentration, and their interaction all significantly influenced (*p* < 0.05) mechanical performance. Both strength and modulus decreased as PBAT content and RA increased, with the highest values of 50 MPa and 2.78 GPa observed for neat PLA 3D-printed at 0° RA, and the lowest values of 15 MPa and 1.05 GPa for 40 wt.% PBAT at 90° RA. In contrast, incorporating PBAT improved impact strength, showing its toughening effect. Meanwhile, no clear trend between impact resistance and RA was observed. The highest impact strength (52.7 kJ/m^2^) was found at 40 wt.% PBAT content and ±45° RA.

## 1. Introduction

Additive manufacturing (AM) is an emerging manufacturing technology encompassing a wide range of processes that produce objects by building them up in a layer-by-layer fashion. AM, often referred to as 3D printing, allows for innovative product design, rapid prototyping, and the combination of multiple materials within a single product, features that would be difficult to achieve using traditional manufacturing methods [1]. Therefore, AM has found applications across a wide variety of fields, including agriculture, engineering, medicine, defense, and the food industry [2].

Currently, the most diffused 3D printing technique utilized both by individual users and industrial players is material extrusion (MEX), also known as fused deposition modeling (FDM) or fused filament fabrication (FFF). MEX has emerged as one of the most reliable and versatile AM techniques owing to its simplicity and relatively low cost of equipment. It relies on thermoplastic feedstock. The most commonly used thermoplastic materials for MEX include poly(lactic acid) (PLA) [3,4], acrylonitrile butadiene styrene (ABS) [5], glycol-modified poly(ethylene terephthalate) (PETG) [6], polyamide (PA) [6,7], and thermoplastic polyurethane (TPU) [8]. Recently, increasing environmental awareness has contributed to biopolymers becoming more attractive alternatives to traditional plastics, with PLA becoming the most widely used material in MEX 3D printing.

PLA is a linear aliphatic thermoplastic polyester synthesized from lactic acid, which is produced through the fermentation of renewable biomass such as corn, sugarcane, and other starch-rich crops [9]. It is a semi-crystalline polymer with a glass transition temperature of 50–70 °C and a melting temperature ranging from 130 to 180 °C. PLA has mechanical properties comparable to commodity plastics such as polystyrene (PS) and poly(ethylene terephthalate) (PET) [10], and its stable melt flow, low shrinkage, and minimal warping make it easy to process using MEX 3D printing. These features greatly contribute to its suitability for additive manufacturing of rigid parts. However, PLA is inherently brittle, which limits its use in applications that need high impact resistance and flexibility unless it is modified or blended [11]. Moreover, there are still just a handful of eco-friendly alternatives for MEX 3D-printing feedstock. In response to growing demand, researchers and industrial players have increasingly turned their attention to PLA-based polymer blends in which ductile, biodegradable plastics are introduced as secondary components to produce green materials with enhanced toughness. Potential modifiers include natural rubber (NR) [12], poly(butylene succinate) (PBS) [13], polycaprolactone (PCL) [14], and poly(butylene adipate-co-terephtalate) (PBAT) [15,16,17].

PBAT, like PLA, is a biodegradable thermoplastic polyester. It is a semi-crystalline polymer with outstanding ductility and is commonly used as a biodegradable substitute for low-density polyethylene (LDPE) [18]. Its application as a standalone material in MEX 3D printing is challenging. Due to its soft nature, it is difficult to extrude through the nozzle, and its intensive shrinking and warping often cause printed parts to delaminate from the build platform. By blending PLA with PBAT, the excellent printability of PLA can be preserved, while PBAT can effectively compensate for PLA’s brittleness. Consequently, PLA/PBAT blends represent an optimal compromise between strength and toughness as shown in the available literature.

Andrzejewski et al. [15] fabricated MEX 3D-printing filaments from PLA/PBAT blends containing 0–30 wt.% PBAT along with 0.5–1 wt.% multifunctional chain extender as a compatibilizer. The authors analyzed the effect of blend composition while also varying the printing speed in the range of 20–60 mm/min. Mechanical testing showed that the presence of an elastomeric PBAT phase increases plastic deformation and Izod impact strength, albeit at the cost of decreased modulus and strength. As for printing speed, increasing the speed led to reduced impact resistance, whereas other mechanical properties remained largely unchanged. Yu et al. [17] prepared filaments with PLA/PBAT weight ratios from 100/0 to 60/40. The highest elongation, 10.15%, was obtained for the blend containing 30 wt.% PBAT, representing a 3.08% increase over neat PLA; however, this improvement was accompanied by decreases in tensile and flexural strength and stiffness. De Melo et al. [19] investigated a commercial blend containing 86 mol% PBAT and 14 mol% PLA, studying the effects of printing temperature (180 to 210 °C) and speed (50 mm/s and 80 mm/s) on mechanical properties. The highest elongation at break (374%) occurred at 210 °C and 50 mm/s, while the maximum tensile strength and modulus of elasticity were achieved at 210 °C and 80 mm/s.

While blending PLA with PBAT is well known to improve toughness, using these blends in MEX 3D printing is still challenging because mechanical performance depends on both blend composition and applied printing parameters. Up until now, the interactive effects between PBAT content and raster orientation, one of the most influential MEX processing parameters, have not yet been systematically and statistically evaluated. In order to address this knowledge gap, PLA/PBAT blends with 0–40 wt.% PBAT content were prepared and characterized for their melt flow behavior and thermal properties to confirm their suitability as MEX 3D-printing feedstock. Subsequently, specimens for tensile and impact tests were 3D-printed using raster angles of 0°, ±45°, and 90°, and the resulting mechanical properties were analyzed using statistical methods in order to quantify the individual and interaction effects of PBAT concentration and raster orientation on the mechanical performance of 3D-printed PLA/PBAT structures.

## 2. Materials and Methods

### 2.1. Materials

For the experiments, Ingeo 3052D grade PLA, with a D-lactide content of ~4%, a *T_g_* range of 55–60 °C, a melting temperature range of 145–160 °C, and a melt flow index of 14 g/10 min (at 210 °C, with a 2.16 kg load), was procured from NatureWorks LLC (Minnetonka, MN, USA). The PBAT, used as a toughening agent for PLA, was a Biopolyester Purge grade pellet obtained from BASF (Ludwigshafen, Germany), and it has a melt flow index of 2.5–4.5 g/10 min (at 190 °C, 2.16 kg).

### 2.2. Blend Preparation

Prior to melt compounding, a 4 h drying step at 80 °C was applied using a WGLL 125 BE dehumidification chamber (Huanghua, Cangzhou, China) in order to remove excess moisture from the components. Neat PLA served as the reference material, and four additional blends were prepared with PBAT contents of 10, 20, 30, and 40 wt.%. Samples were designated based on PBAT content as P0, P10, P20, P30, and P40.

The dried PLA and PBAT granules were manually dry-mixed and fed into a LabTech LTE 20-44 twin-screw extruder (Samut Prakan, Thailand). The barrel heating zones were set to 155–155–160–160–165–170–175–180–180–185–185 °C from the feeder to the die, and the screw rotational speed was 30 rpm. The extruded strands were cooled in a water bath and subsequently ground using a LabTech LZ-120 pelletizer (Samut Prakan, Thailand). The resulting pellets were used for filament fabrication after an additional drying step under the same conditions described above.

### 2.3. Filament Fabrication

The biopolymer blends were processed into filaments using a 3devo Precision 450 filament maker extruder (Utrecht, The Netherlands). Filaments with a diameter of 1.75 ± 0.05 mm were produced. The machine parameters were as follows: heating zone temperatures of 170–190–185–170 °C from the feeder to the die, and a screw rotational speed of 3.5 rpm.

### 2.4. Three-Dimensional Printing Process

Rectangular- and dumbbell-shaped specimens were fabricated using a Craftbot Plus desktop MEX 3D printer (Budapest, Hungary). The tensile specimens corresponded to the 1BA type geometry of the ISO 527-2 standard [20], while rectangular bars with dimensions of 80 × 10 × 4 mm^3^ were prepared for impact testing. G-codes were generated from STL files using the SuperSlicer software (version: 2.5.59.13). The printing parameters applied during specimen fabrication were chosen based on literature data [17] and preliminary experiments and are summarized in Table 1. Additionally, an extrusion rate (k) equal to 1.03 was used.

In order to investigate the effect of linear infill orientation, three raster angles (RAs) were employed. The infill patterns were oriented relative to the longest dimension of the specimens: an infill parallel to the longest side was assigned RA = 0°, while a perpendicular pattern corresponded to RA = 90°. Additionally, an alternating ±45° raster orientation was also tested. In order to distinguish specimens with different RAs, the raster angle was included as a suffix in the sample designation. For example, a PLA/PBAT blend with a 90/10 weight ratio 3D-printed with a ±45° raster angle is denoted as “P10_45”.

### 2.5. Characterization

Thermal properties and crystallinity of the samples were analyzed using a DSC 200 F3 differential scanning calorimetric (DSC) apparatus (Netzsch, Selb, Germany). Measurements followed a heat–cool–heat cycle between 30 and 200 °C at 5 °C/min under a nitrogen atmosphere with a flow rate of 20 mL/min. The first heating sequence served the purpose of erasing the thermal history of the samples, and the second heating cycle was evaluated to determine the degree of crystallinity (*X_c_*), glass transition temperature (*T_g_*), cold crystallization temperature (*T_cc_*), and melting temperature (*T_m_*) of PLA. The degree of crystallinity was calculated using Equation (1):

(1) $$X_{cPLA}=\frac{\left|∆H_{m}-∆H_{cc}\right|}{∆H_{mPLA}^{\infty }\cdot \omega_{PLA}}\times 100$$

where Δ*H_m_* is the melting enthalpy, Δ*H_cc_* is the enthalpy of cold crystallization, $∆H_{mPLA}^{\infty }$ is the theoretical melting enthalpy of 100% crystalline PLA (taken as 93 J/g based on the literature [21,22], and *ω_PLA_* is the weight fraction of PLA in the sample.

To analyze the chemical and structural properties of the fabricated samples, Fourier transform infrared spectroscopy (FTIR) was used. Measurements were performed on a Frontier FT-IR spectrometer (PerkinElmer, Waltham, MA, USA) equipped with a universal attenuated total reflectance (UATR) accessory with a ZnSe single-bounce crystal. Spectra were collected in the 4000 to 600 cm^−1^ wavenumber range with 64 scans at 4 cm^−1^ resolution.

The melt flow index (MFI) was measured using a Ceast 7026 modular melt flow apparatus (Pianezza, Italy) following the ISO 1133 standard [23], with a 2.16 kg load at 230 °C, which is the same as the nozzle temperature during 3D printing. The results represent the average values of five consecutive measurements.

Cryogenic fracture surfaces of the samples were examined using scanning electron microscopy (SEM) with a Crossbeam 350 apparatus (Zeiss, Oberkochen, Germany) operated at an accelerating voltage of 5 kV. A thin layer of gold was applied to the surface using an Emitech SC7620 mini sputter coater machine (Quorum Technologies, Laughton, UK) to prevent sample charging.

The density of the filaments was determined according to Archimedes’ principle, following the standard ISO 1183-1 [24], using a Kern ABP100-5DM analytical balance (Kern & Sohn, Balingen, Germany) equipped with a YDB03 density measurement kit (Kern & Sohn, Balingen, Germany). The results represent the average values of three consecutive measurements. Void content (%) in the blends was determined by normalizing the difference between the theoretical and experimental densities by the theoretical density, whereas the theoretical density was computed using the inverse rule of mixture.

Tensile tests were performed with an Instron 5582 universal testing machine (Norwood, MA, USA) equipped with a 10 kN load cell, following the ISO 527 standard [20]. The clamping distance was 58 mm. Specimens were tested at 1 mm/min up to 0.3% strain to determine Young’s modulus, followed by 5 mm/min until fracture. Impact strength was measured according to ISO 179 [25] using a 6545-type pendulum impact tester (Ceast, Torino, Italy) with a 15 J impact hammer and a 62 mm span. The results reported are the averages of four consecutive measurements in both cases. A statistical analysis was performed to evaluate the influence of PBAT content and RA on the investigated mechanical properties of the 3D-printed samples. A two-way analysis of variance (ANOVA) with replication was conducted using the measured values as the response variable and PBAT content and raster angle as fixed factors.

The significance of data differences was statistically analyzed by one-way ANOVA. Tukey’s honest significance test with a 95% confidence level was used to identify which data groups were significantly different from others.

## 3. Results and Discussion

### 3.1. Differential Scanning Calorimetry

**Figure 1:**
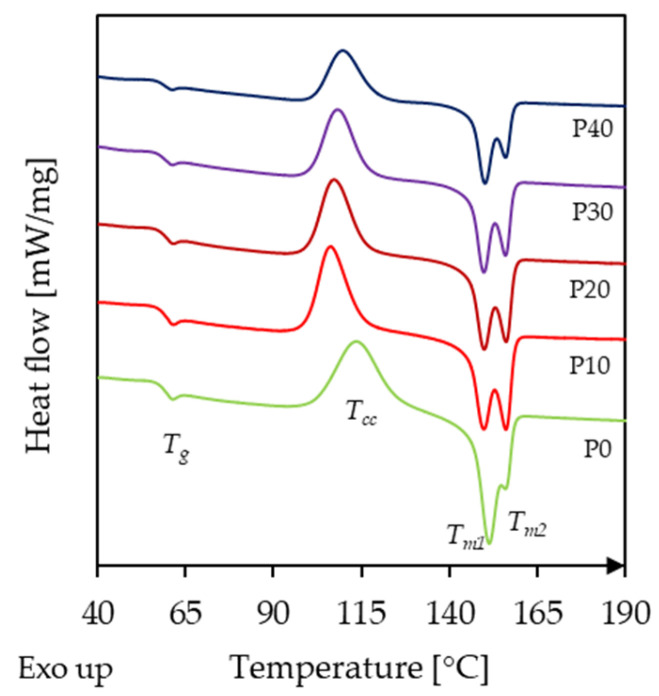
DSC curves of neat PLA and the PLA/PBAT biopolymer blends containing different amounts of PBAT.

The second heating curves recorded during the DSC measurements are displayed in Figure 1, and the corresponding data are summarized in Table 2. Neat PLA exhibited a glass transition temperature of 57.0 °C, marked by a shift in the baseline, which is in good agreement with literature values [26,27]. This was followed by cold crystallization, represented by an exothermic peak at 113.7 °C. Melting occurred in two steps (151.3 °C and 155.9 °C), manifested as a double endothermic peak, which is commonly attributed to melt recrystallization of PLA during heating [28].

With the incorporation of PBAT, the *T_g_* of PLA decreased slightly due to the plasticization effect of PBAT, reaching its lowest value (56.7 °C) for the P30 sample. The small magnitude of this decrease suggests limited interfacial interaction between PLA and PBAT. A more prominent change was observed for *T_cc_*: it decreased by 7.4 °C in the presence of 10 wt.% PBAT (106.3 °C) but then gradually increased with further PBAT addition, reaching 109.6 °C for the P40 sample. Such behavior, an initial drop in the cold crystallization temperature at low concentrations of the secondary component followed by an increase at higher loadings, is frequently reported for binary PLA-based systems, including blends and composites [17,29]. The decrease in *T_cc_* can be attributed to the mobility-enhancing effect of PBAT, which enables crystallization to occur earlier during heating. In addition, PBAT, as later shown in SEM micrographs, forms finely dispersed domains in PLA, which might also facilitate nucleation. At higher PBAT concentrations, however, PBAT hinders PLA chain diffusion, thereby increasing *T_cc_*. Rodriguez et al. [30] reported similar trends when examining PLA/PBAT mixtures of varying composition. In their study, neat PLA exhibited a *T_cc_* of 107.2 °C, which decreased to 102 °C at 10 wt.% PBAT and increased again to 106 °C at 50 wt.%.

The melting temperatures remained largely unchanged, but the relative prominence of the two melting peaks shifted noticeably. For neat PLA, the first melting peak was dominant; at 10 wt.% PBAT, the two peaks became nearly equal; above this content, the first melting point became more dominant again. This trend correlates well with the observed changes in cold crystallization temperature. As widely reported, the melting behavior is related to the temperature range in which crystallites are formed. Crystallites formed at higher temperatures (110–130 °C) tend to produce a more prominent *T_m_*_1_, whereas crystallites formed at lower temperatures (80–110 °C) favor *T_m_*_2_ [31]. Thus, the observed changes in cold crystallization temperature and the relative melting peak intensities are consistent.

### 3.2. Fourier Transform Infrared Spectroscopy

**Figure 2:**
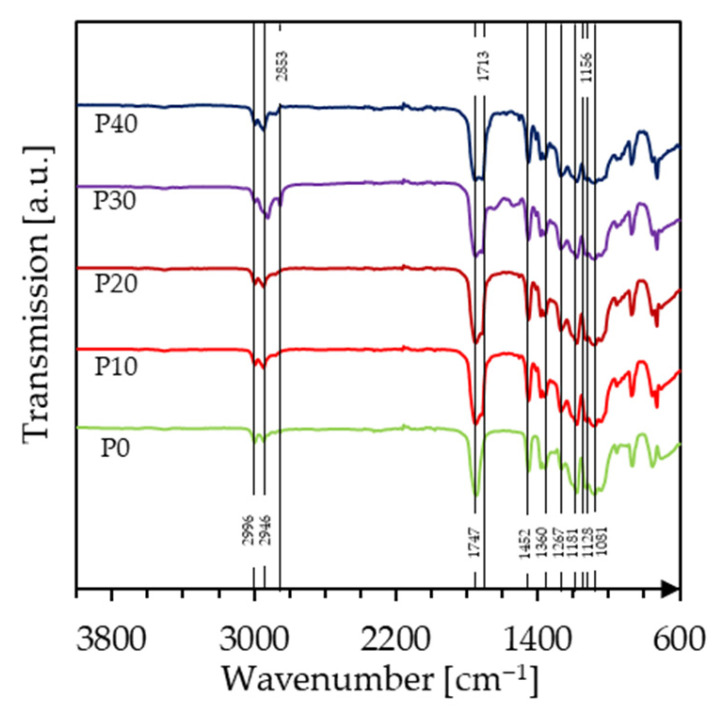
FTIR spectra of PLA and the PLA/PBAT biopolymer blends containing different amounts of PBAT.

In order to examine potential chemical interactions in the PLA/PBAT blends, FTIR experiments were performed. As shown in Figure 2, pure PLA exhibits multiple absorption bands in the region of 3000–2900 cm^−1^, corresponding to the asymmetric and symmetric stretching vibrations of aliphatic C-H bonds. Specifically, the peaks at 2996 cm^−1^ and 2946 cm^−1^ are related to the stretching vibrations of CH_3_ groups with minor CH_2_ contributions [32]. The strong absorption band at 1747 cm^−1^ can be attributed to the C=O ester carbonyl stretching vibration, confirming the polyester structure of PLA [33]. The band at 1452 cm^−1^ is associated with the asymmetric bending vibration of methyl (CH_3_) groups, while the band at 1360 cm^−1^ represents the C-O-H bending vibrations. Bands at 1267 cm^−1^ can be attributed to the C-O bending, while those at 1181 cm^−1^ and 1128 cm^−1^ correspond to the C-O-C stretching vibration of the ester linkage.

With the addition of PBAT, the FTIR spectrum exhibited additional absorption bands and changes in the intensity of already existing ones. The absorption band at 2946 cm^−1^, also observed for pure PLA, increased in intensity with higher PBAT concentration, which can be ascribed to the greater concentration of aliphatic methylene (CH_2_) groups in the PBAT backbone. A new absorption band at 2853 cm^−1^ appeared, corresponding to the symmetric stretching of CH_2_ groups, characteristic of PBAT [34]. Similar to PLA, PBAT also displays a major band corresponding to ester carbonyl stretching vibrations, appearing at a slightly lower wavenumber (1713 cm^−1^) compared to PLA due to differences in the chemical environment. The peak at 1267 cm^−1^ also became more prominent with increasing PBAT concentration, indicating C-O stretching in the ester linkage. At 1156 cm^−1^, the transmittance of PLA decreased with the addition of PBAT, marking the C-O-C stretching vibrations of both adipate and terephthalate ester units.

The positions of the bands corresponding to PLA and PBAT remained essentially unaltered regardless of blend composition, indicating that no primary chemical interactions occur between the components. This observation is in good agreement with the DSC results.

### 3.3. Melt Flow Behavior

**Figure 3:**
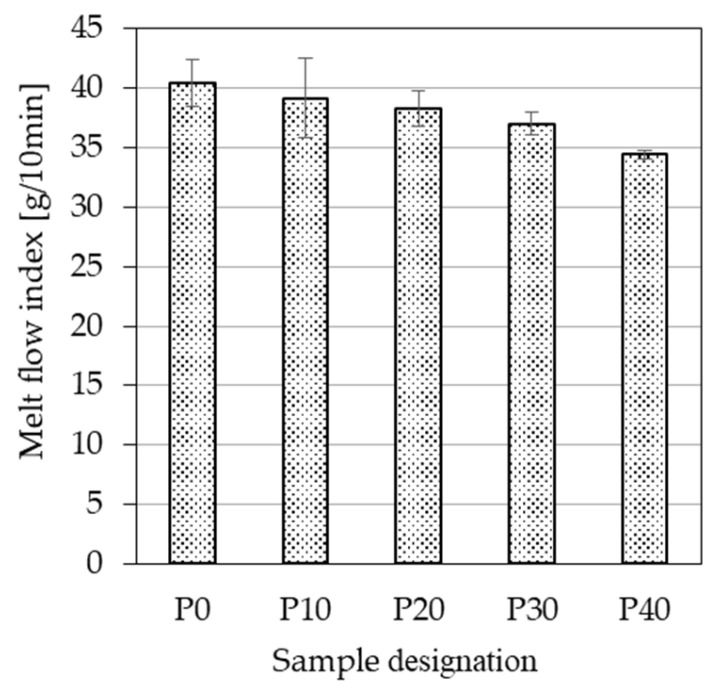
The melt flow index of neat PLA and the PLA/PBAT biopolymer blends containing different amounts of PBAT.

Figure 3 displays the measurement results of the melt flow index tests. The rheological properties of polymers are crucial in MEX 3D printing. Suitable melt rheology facilitates smooth extrusion through the nozzle and helps prevent shape instability and defect formation in the printed parts. In industrial practice, MFI is widely used to characterize the flow uniformity of thermoplastic materials. In the context of MEX 3D printing, MFI not only influences the choice of printing parameters but also affects interlayer bonding. Therefore, assessing this property is essential for understanding both processing behavior and final part quality.

Neat PLA exhibited a melt flow index of 40.4 g/10 min. This value decreased to 39.2 g/10 min with 10 wt.% PBAT and continued to decline as PBAT content increased, reaching 34.4 g/10 min at 40 wt.%. Evidently, PBAT introduces greater resistance to viscous flow than PLA, likely due to its higher degree of chain entanglement. Similar results were reported by Duan et al. [35] when investigating PLA/PBAT/epoxy-functional polymer blends. At a 70/30 PLA/PBAT ratio, the authors measured an MFI of 32.8 g/10 min, which decreased to 15.8 g/10 min at a 30/70 ratio.

Overall, the presence of PBAT clearly reduces the melt flow index of PLA; however, since the rate of decrease remains below 15% even at 40 wt.% PBAT content, it is not expected to significantly interfere with processability.

### 3.4. SEM Analysis

**Figure 4:**
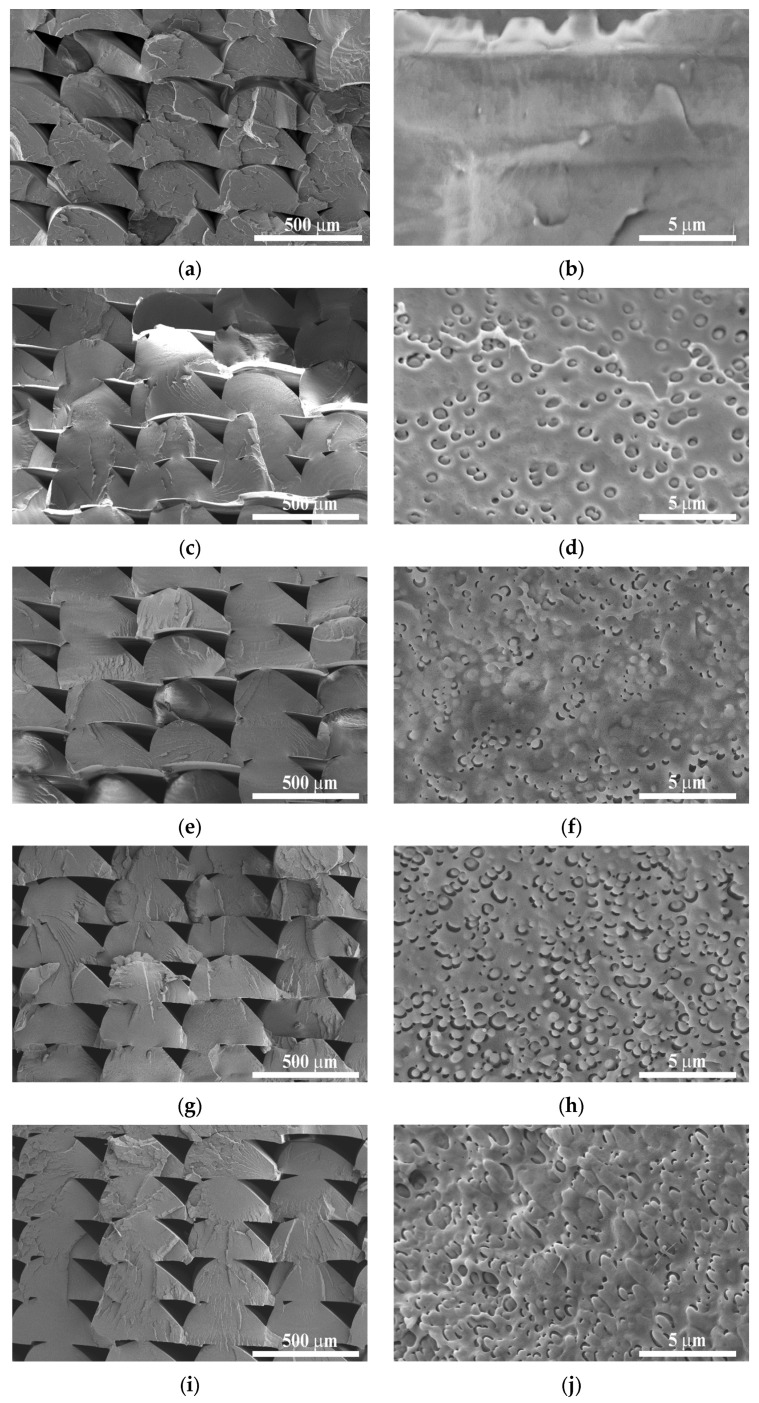
Scanning electron microscopic images of samples P0_0 (**a**,**b**), P10_0 (**c**,**d**), P20_0 (**e**,**f**), P30_0 (**g**,**h**), and P40_0 (**i**,**j**) at different magnifications.

The microstructure morphologies of the 3D-printed specimens were examined by means of SEM (Figure 4). All samples exhibit a typically brittle fracture surface, which is reasonable considering that the fractures were prepared cryogenically. In each sample, bonding between adjacent beads occurs primarily at their contact surfaces, and voids of considerable size with a quasi-diamond shape can be clearly observed (Figure 4a,c,e,g,i). The size and shape of these voids are relatively uniform across the samples P0, P10, P20, and P30, with widths of ~100 μm, indicating that the introduction of the PBAT component at these concentrations does not negatively influence processability. At 40 wt.% PBAT, however, the voids appear slightly larger. Since bead-to-bead bonding is largely governed by melt viscosity during the 3D printing process, it can be assumed that the presence of PBAT increases the viscosity of the blend [36]. This is consistent with the melt flow index results. Higher viscosity slows molecular diffusion at the interface and thus reduces the coalescence rate between adjacent beads.

The fracture surfaces at higher magnification are shown in Figure 4b,d,f,h,j. Neat PLA (Figure 4b) exhibits a rather smooth, homogeneous fracture surface with essentially no pores or voids. In contrast, all PBAT-containing blends display a typical sea-island morphology, where globular PBAT domains of <1 μm are uniformly dispersed within the continuous PLA matrix. This domain diameter is generally sufficient to allow for effective toughening in elastomer-modified plastics [37]. Based on the SEM micrographs, PBAT globule size appears to be independent of PBAT concentration, with the exception of 40 wt.% loading, where slight coalescence of PBAT domains is visible. The observed morphologies and domain sizes are consistent with those reported for PLA/PBAT biopolymer blends in the literature [38,39,40]. Clear phase separation with thin voids between the PLA and PBAT phases is evident in all blends, suggesting limited interfacial adhesion between the two polyesters. This is in good agreement with the results of the DSC and FTIR measurements. The presence of such voids typically adversely affects the mechanical performance of polymeric materials, especially strength and stiffness. However, in toughened systems, voids may also facilitate energy-dissipating mechanisms, including void growth, crack deflection, and plastic deformation, which, on the other hand, may improve impact toughness.

Note that the SEM analysis presented in the current study is intended to provide a qualitative assessment to support the observations regarding inter-bead bonding and phase separation relevant to printed structures. While the evolution of blend morphology and the quantitative characterization of PBAT phase domains, such as domain size distribution or morphology evolution, are considered important factors in immiscible polymer blends, these are beyond the scope of the present work.

### 3.5. Density

**Figure 5:**
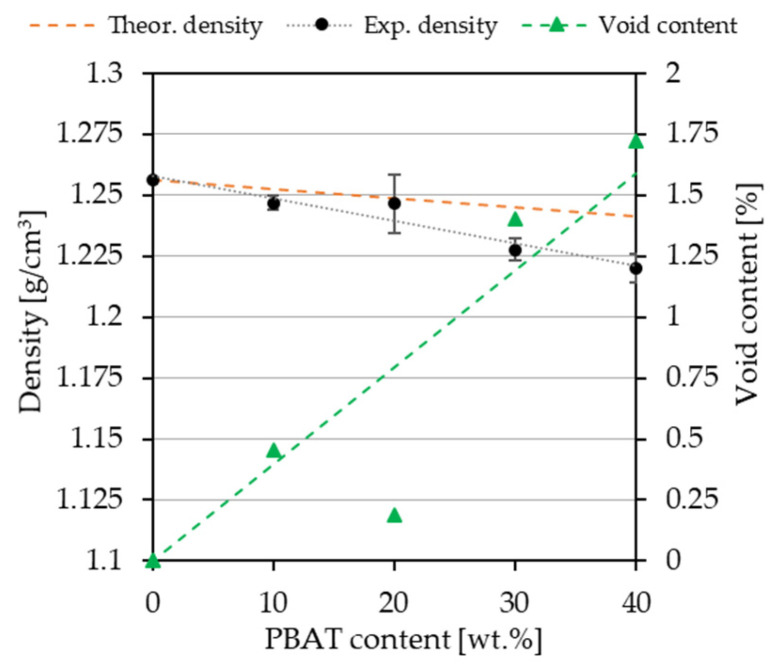
Density and void content of PLA and the PLA/PBAT biopolymer filaments containing different amounts of PBAT.

The results of the theoretical and experimental densities of the filaments, as depicted in Figure 5, fluctuated within a small range from 1.220 to 1.256 g/cm^3^. Neat PLA exhibited the highest value of density, while the density of the P40 blend containing 40 wt.% PBAT was the lowest, demonstrating that density decreased with increasing PBAT concentration. The void content of the blends was found to increase with increased PBAT loading, peaking at 40 wt.% PBAT loading (1.72%), which can be ascribed to poor interfacial adhesion between the components, consistent with the DSC, FTIR, and SEM observations. These findings are also in good agreement with observations described in other studies [41].

### 3.6. Tensile Mechanical Properties

#### 3.6.1. Tensile Strength

**Figure 6:**
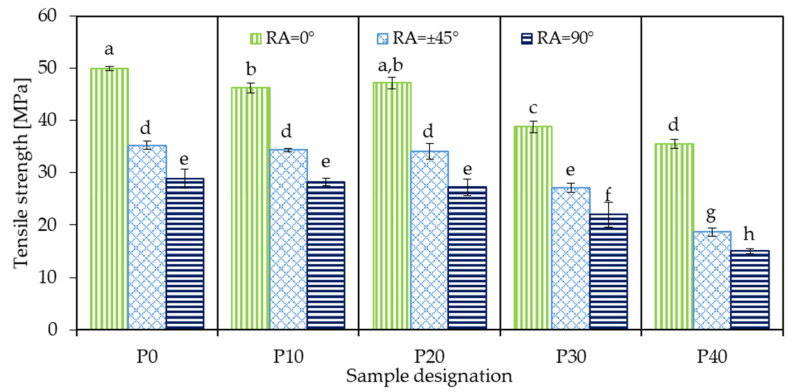
Tensile strength of 3D-printed neat PLA and PLA/PBAT biopolymer blends (identical letters above the bars mean no significant difference according to Tukey’s honest significance test).

The tensile strength of the samples is displayed in Figure 6. The neat PLA with an infill orientation parallel to the length of the specimen (P0_0) exhibited the highest tensile strength (50.0 MPa). With increasing RA, the strength of PLA gradually decreased to 35.2 MPa for P0_45 and to 28.9 MPa for the P0_90 samples. This suggests that an infill orientation parallel to the tensile load is the most beneficial. This is due to the fact that when beads are oriented in the same direction as the tensile load, the inherently weaker inter-bead regions are not subjected to mechanical loads, but rather the bulk beads bear the load [42]. With increasing RA, the role of inter-bead regions in load bearing becomes more and more prominent, resulting in reduced load-bearing capacity. This kind of behavior in MEX 3D-printed structures is well-documented in the literature. For example, Kargar and Ghasemi-Ghalebahman [43] conducted experiments with different printing parameters using the Taguchi method on a carbon-fiber reinforced filament and analyzed the effects of multiple printing parameters, including raster angle. At a 0° raster angle, a maximum mechanical stress of 59.3 MPa was measured during the tensile test, while at ±45° it decreased to 53.2 MPa and drastically decreased to 38.3 MPa at 90°. Ramírez-Prieto et al. [44] conducted research with a commercially available PLA filament that contained 2–4% calcium carbonate, testing the mechanical properties of samples prepared at different raster angles. The 0° samples had the highest average ultimate tensile strength of 38.2 MPa, followed by the ±45° samples (35.8 MPa), and finally the 90° samples with 31.5 MPa, although alternating with a 0° layer.

Incorporating 10 wt.% PBAT into PLA (P10) resulted in reduced tensile strength. At 0° RA, the initial 50.0 MPa dropped to 46.3 MPa, and this value further decreased to 34.4 MPa and 28.3 MPa at ±45° and 90° RA, respectively. The reduced strength can be attributed to the inherently softer nature of PBAT compared to PLA, as well as the limited interfacial adhesion between them. Note that the relative drop with increasing RA was less drastic compared to the P0 sample; instead of 30% and 42% decrease at ±45° and 90°, respectively, it was only 26% and 39%, indicating better inter-bead adhesion in the presence of 10 wt.% PBAT.

Yu et al. [17] investigated the effect of PBAT content on PLA/PBAT blends; however, this was limited to ±45° RA. The authors also found a decrease in tensile strength along with an increase in PBAT ratio relative to the PLA. At 10 wt.% PBAT content, the authors found a drop from 39 MPa to 29 MPa in tensile strength.

Increasing the PBAT content to 20 wt.% (sample P20) did not result in a significant alteration in tensile strength compared to P10, regardless of the applied RA. However, a further increase in PBAT concentration resulted in a considerable decrease. At 30 wt.%, the tensile strength of the samples dropped by 6–8 MPa at all raster angles, while at 40 wt.% the strength values bottomed out at 35.5 MPa, 18.7 MPa, and 15.0 MPa at 0°, ±45°, and 90° RA, respectively. It can be assumed that with increasing concentration, the previously finely dispersed, small PBAT domains became larger due to coalescence and acted as stress concentration sites, triggering early cavitation and shear yielding. These findings are consistent with those reported in the study by Mathew et al. [45], where the authors performed a comparative analysis on PLA/PBAT mixtures at different PBAT ratios. In that case, the tensile strength of 3D-printed PLA decreased from ~48 MPa to ~28 MPa when 40 wt.% PBAT was incorporated.

**Figure 7:**
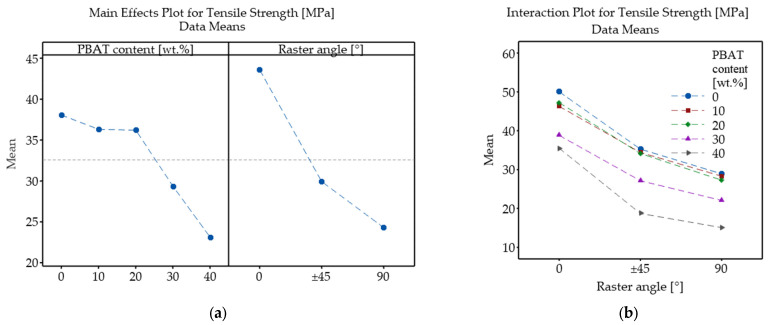
Main effects (**a**) and interaction plot (**b**) showing the influence of PBAT content and raster angle on tensile strength of PLA/PBAT samples.

Figure 7 presents the main effects and interaction plots of tensile strength as a function of RA and PBAT content. According to the main effects plot (Figure 7a), PBAT contents up to 20 wt.% do not significantly reduce tensile strength; however, samples P30 and P40 exhibit considerably lower strength values. Increasing the raster angle has a similarly pronounced negative effect. The interaction plot in Figure 7b shows how RA influences tensile strength at each PBAT level. All curves exhibit a similar general trend, and the curves corresponding to P0, P10, and P20 are nearly overlapping, indicating little-to-no interactions at these compositions. On the other hand, the curves corresponding to P30 and P40 shifted to a lower mean strength values and are also not perfectly parallel to the other curves, suggesting a soft interaction.

The ANOVA results (Table 3) indicate that PBAT content, RA, and their interaction all had a statistically significant effect on tensile strength. Among the two variables, raster angle was more dominant, as evidenced by its considerably higher F-value (1399.67) compared to that of PBAT loading (337.77). Although the interaction produced the smallest F-value (4.55), it still exceeded the corresponding critical value, confirming that PBAT content and raster angle do not act independently with respect to tensile strength.

#### 3.6.2. Young’s Modulus

**Figure 8:**
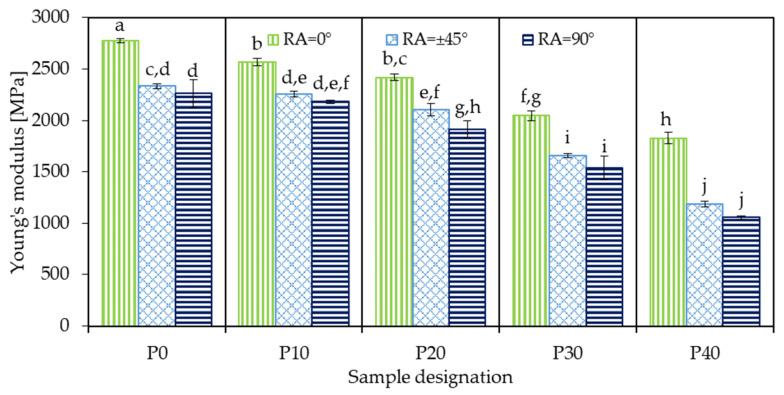
Young’s modulus of 3D-printed neat PLA and PLA/PBAT biopolymer blends (identical letters above the bars mean no significant difference according to Tukey’s honest significance test).

Figure 8 shows the Young’s modulus of 3D-printed neat PLA and its PBAT-containing biopolymer blends manufactured using different raster angles. Sample P0_0 (neat PLA 3D-printed at 0° RA) exhibited the highest modulus of 2778 MPa, which is comparable to values reported in the literature for PLA processed by conventional manufacturing techniques such as injection molding [9]. Increasing the raster angle to ±45° resulted in a considerable reduction to 2331 MPa for sample P0_45, while further increasing the raster angle to 90° led to an additional, but less prominent, drop to 2262 MPa (sample P0_90). It is commonly reported in the literature that an infill orientation parallel to the tensile load (RA = 0°) typically results in the highest stiffness, while deviations in raster angle tend to reduce the modulus. For instance, Patel et al. [46] conducted a full-factorial experiment on 3D-printed PETG samples, varying several process parameters, including RA. Their results showed that specimens 3D-printed at 90° RA exhibited the lowest modulus, followed by those fabricated at ±45° RA, while the highest stiffness was observed for samples prepared at 0° RA. This trend was consistent across different layer thicknesses (0.2, 0.3, and 0.4 mm) and printing speeds (40 mm/s, 50 mm/s, and 60 mm/s).

This kind of behavior is attributed to the increasing influence of inter-bead regions on the mechanical response as the raster angle rotates away from the loading direction. Inter-bead regions inherently possess a lower modulus than the bulk material for multiple reasons. Firstly, the deposited beads exhibit an elliptical cross-section (as also shown in the SEM images in Figure 4), and the resulting reduced contact area between neighboring beads decreases the effective load-bearing cross-section. Secondly, due to limited cohesion between the deposited beads, the inter-bead regions are mechanically weaker than the continuous bead material.

The incorporation of PBAT into PLA also resulted in softer behavior, as evidenced by the consistently decreasing modulus with growing PBAT content. This is attributed to the rubbery, flexible nature of the PBAT domains, and such reductions in stiffness are commonly reported in elastomer-toughened polymer blends. As expected, the modulus decreased consequently with increasing PBAT loading. Accordingly, the lowest Young’s modulus, 1055 MPa, was observed for sample P40_90.

**Figure 9:**
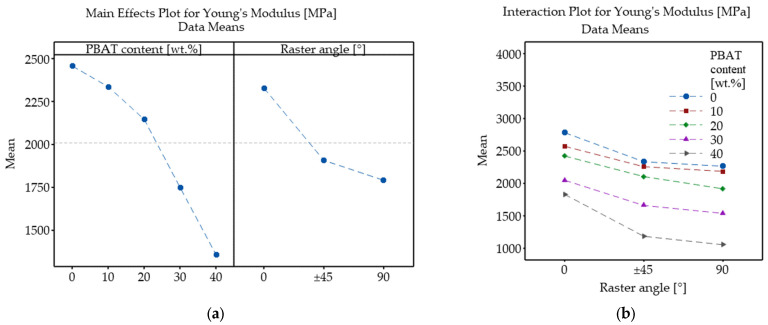
Main effects (**a**) and interaction plot (**b**) showing the influence of PBAT content and raster angle on Young’s modulus of PLA/PBAT samples.

The impact of PBAT content and raster angle on the Young’s modulus of the MEX 3D-printed PLA/PBAT samples was evaluated through two-way ANOVA with replication, and the corresponding main effects and interaction plots are presented in Figure 9. As shown in Figure 9a, Young’s modulus monotonously decreased with increasing PBAT content from 0 wt.% to 40 wt.%. Increasing the raster angle exhibited a similar but less significant effect. The interaction plot in Figure 9b shows how RA affects Young’s modulus at each PBAT level. All curves exhibit a similar general trend; however, due to them not being perfectly parallel, it can be concluded that there is a soft interaction between the two factors. No line crossings were observed, confirming that the ranking of raster angle effects does not change with composition.

The ANOVA results (Table 4) indicate that both PBAT content, RA, and their interaction have statistically significant effects on Young’s modulus, as all *p*-values were far below 0.05. Among these, PBAT concentration was the most dominant factor, with the highest F-value of 682.71, followed by raster angle (442.57). The interaction, although much smaller in magnitude (7.58), is still significant.

### 3.7. Impact Strength

**Figure 10:**
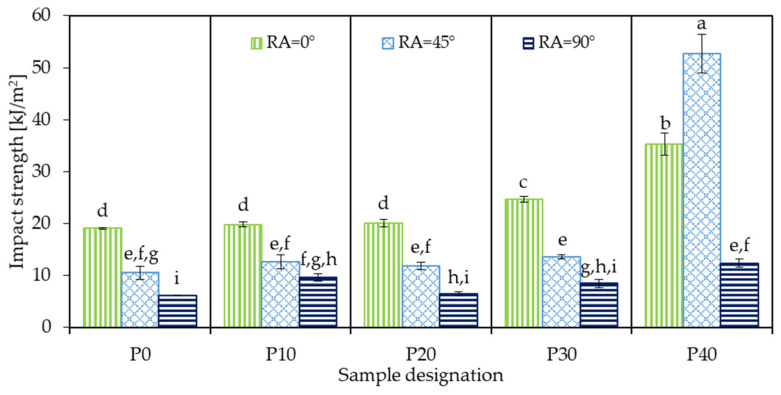
Charpy impact strength of 3D-printed neat PLA and PLA/PBAT biopolymer blends (identical letters above the bars mean no significant difference according to Tukey’s honest significance test).

The Charpy impact strength results of 3D-printed neat PLA and PLA/PBAT biopolymer blends are presented in Figure 10. Samples P0_0, P0_45, and P0_90 exhibited impact strength values of 19.1, 10.5, and 6.2 kJ/m^2^, respectively. As RA increases, the impact toughness of neat PLA gradually decreases. Similar trends have been reported by other authors [47,48] and are typically explained by the continuous beads at 0° RA, situated perpendicular to the mechanical load, bridging the impact zone, thereby resisting bending and delaying fracture.

Interestingly, the impact strength of the sample 3D-printed at 0° exceeded the values reported in the literature for bulk, injection-molded PLA samples [9], which may be attributed to intensive molecular orientation induced during bead deposition in the MEX 3D-printing process. Although only a limited number of studies report un-notched Charpy impact strength for MEX 3D-printed PLA, Mukoroh et al. [49] measured an impact toughness of 11.1 kJ/m^2^ for PLA samples printed at ±45° RA, which is in good accord with the present results.

The addition of PBAT generally improved impact resistance, although no clear monotonic trend was observed. For samples 3D-printed at 0° RA, a slow but steady increase in toughness was found up to 30 wt.% PBAT content, after which a considerable jump from 24.7 kJ/m^2^ to 35.3 kJ/m^2^ occurred. Meanwhile, samples 3D-printed at ±45° and 90° showed little-to-no change up to 30 wt.% PBAT concentration, fluctuating in the range of 10–14 kJ/m^2^ and 6–10 kJ/m^2^, respectively. However, at 40 wt.% PBAT concentration, a considerable increase was observed, with sample P40_45 reaching the highest impact strength of 52.7 kJ/m^2^, outperforming even the corresponding 0° sample, which was not typical for any other PLA/PBAT blend.

While this behavior may seem contradictory, it can be explained by the different fracture mechanisms typical for brittle and toughened materials. For brittle polymers, including PLA and its blends with low PBAT content, fracture occurs through rapid crack propagation with little-to-no plastic deformation. When 3D printing is performed at 0° RA, cracks must cut through the solid beads rather than propagate along the mechanically weaker inter-bead regions, which increases the energy required for fracture. Accordingly, a 0° RA typically exhibits the best impact toughness for these materials.

On the other hand, in elastomer-toughened systems, such as PLA with sufficiently high PBAT content, impact energy is dissipated through extensive plastic deformation mechanisms, including shear banding, ligament stretching, and crack deflection, rather than pure bending. At a ±45° RA, shear stresses are dominant during the impact, promoting plastic deformation processes, while crack paths also become more tortuous due to the alternating filament directions, thereby increasing the energy required for fracture. Several studies [50,51,52] have reported similar findings, concluding that an alternating RA of 45° provides optimal resistance against dynamic impact loading.

**Figure 11:**
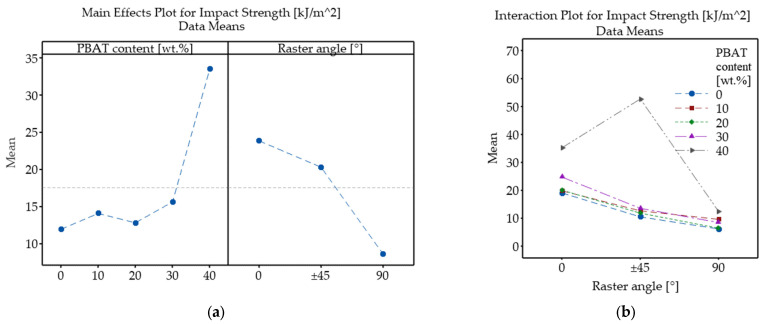
Main effects (**a**) and interaction plot (**b**) showing the influence of PBAT content and raster angle on the impact strength of PLA/PBAT samples.

Figure 11 presents the main effects and interaction plots regarding the effect of PBAT content and raster angle on the impact strength of MEX 3D-printed PLA/PBAT samples. As shown in Figure 11a, PBAT content has a nonlinear effect on impact toughness; up to 30 wt.% loading, only slight variations are observed, followed by a noticeable improvement at 40 wt.% PBAT concentration. The interaction plot (Figure 11b) shows barely any interactions between the examined factors for PBAT contents between 0 and 30 wt.%, as evidenced by the overlapping curves. At 40 wt.% PBAT concentration; however, there is a large peak at ±45° RA, followed by a sharp drop at 90°, which indicates a strong interaction between composition and raster orientation.

Based on the ANOVA results (Table 5), it can be concluded that PBAT loading, RA, and their interaction all have statistically significant effects on impact strength. Raster angle emerged as the factor with the strongest effect (F = 751.38), although PBAT content’s impact was also very strong (F = 579.31) on toughness. Even though the interaction exhibited the smallest F-value (F = 167.45), it was nonetheless statistically significant and substantially stronger than the interaction effects observed for any of the other investigated mechanical properties (tensile strength and Young’s modulus).

## 4. Conclusions

This study investigated the effect of PBAT content and raster orientation on the mechanical performance of material extrusion 3D-printed PLA/PBAT biopolymer blends. The concentration of PBAT was varied in the range of 0 to 40 wt.%, while raster angles of 0°, ±45°, and 90° were examined. The PLA/PBAT blends were characterized for their thermal and melt flow behavior, and the 3D-printed objects prepared from those were analyzed for their morphological and mechanical properties. Melt flow index measurements showed a slight decrease in flow rate with increasing PBAT content, which can be attributed to the higher viscosity of PBAT compared to PLA. This also led to reduced inter-bead contact surface area, as supported by SEM micrographs. Differential scanning calorimetry revealed a nearly constant *T_g_* for PLA regardless of PBAT content, indicating limited interfacial adhesion between the polymers, which is consistent with the FTIR results and the phase separation observed in the SEM images. The tensile strength of the 3D-printed PLA/PBAT specimens decreased with both increasing raster angle and PBAT content, with raster angle being the more influential factor, and considerable strength reduction taking place above 20 wt.% PBAT content. Young’s modulus also decreased with higher PBAT content and raster angles, with composition being the more dominant factor in this case. Considering the Charpy impact strength, it decreased with increasing raster angle, while PBAT addition generally improved toughness, most notably at 40 wt.% PBAT loading, where the ±45° samples exhibited the highest values. ANOVA analysis confirmed that PBAT content, raster angle, and their interaction all significantly affect impact performance.

## Figures and Tables

**Table 1:** Parameters of the 3D printing process.

Parameter [Unit]	Value
Nozzle temperature [°C]	230
Nozzle diameter [mm]	0.8
Build platform temperature [°C]	60
Printing speed [mm/s]	50
Layer thickness [mm]	0.2
Infill density [%]	100
Bead width [mm]	0.84
First layer thickness [mm]	0.4
First layer width [mm]	0.96

**Table 2:** DSC data of neat PLA and the PLA/PBAT biopolymer blends containing different amounts of PBAT.

Designation	*T_g_* [°C]	*T_cc_* [°C]	*T_m_*_1_ [°C]	*T_m_*_2_ [°C]	Δ*H_cc_* [J/g]	Δ*H_m_* [J/g]	*X_c_* [%]
**P0**	57.0	113.7	151.3	155.9	3.9	4.0	4.3
**P10**	57.0	106.3	149.9	156.1	3.5	3.6	4.3
**P20**	56.9	107.3	149.8	156.2	3.2	3.3	4.4
**P30**	56.7	108.2	150.2	156.0	2.9	3.0	4.6
**P40**	56.8	109.6	151.3	155.9	2.3	2.5	4.5

**Table 3:** ANOVA results of the tensile strength of 3D-printed PLA and PLA/PBAT samples (SS—Sum of Squares; dF—Degrees of Freedom; MS—Mean Square; F crit—Critical F-value).

Source	SS	dF	MS	F	*p*-Value	F Crit
Raster angle	3925	2	1962.7	1399.67	0.0000	3.20
PBAT content	1895	4	473.7	337.77	0.0000	2.58
Interaction	51	8	6.4	4.55	0.0004	2.15
Within	63	45	1.4			
Total	5934	59				

**Table 4:** ANOVA results of Young’s modulus of 3D-printed PLA and PLA/PBAT samples (SS—Sum of Squares; dF—Degrees of Freedom; MS—Mean Square; F crit—Critical F-value).

Source	SS	dF	MS	F	*p*-Value	F Crit
Raster angle	3,197,439	2	1,598,719.5	442.57	0.0000	3.20
PBAT content	9,864,726	4	2,466,181.5	682.71	0.0000	2.58
Interaction	218,969	8	27,371.1	7.58	0.0000	2.15
Within	162,556	45	3612.3			
Total	13,443,690	59				

**Table 5:** ANOVA results of impact strength of 3D-printed PLA and PLA/PBAT samples (SS—Sum of Squares; dF—Degrees of Freedom; MS—Mean Square; F crit—Critical F-value).

Source	SS	dF	MS	F	*p*-Value	F Crit
Raster angle	2521	2	1260.3	751.38	0.0000	3.20
PBAT content	3887	4	971.7	579.31	0.0000	2.58
Interaction	2247	8	280.9	167.45	0.0000	2.15
Within	75	45	1.7			
Total	8730	59				

## Data Availability

The raw data supporting the conclusions of this article will be made available by the authors on request.

## Abbreviations

The following abbreviations are used in this manuscript:

ABS	Acrylonitrile Butadiene Styrene
AM	Additive Manufacturing
ANOVA	Analysis of Variance
DSC	Differential Scanning Calorimetry
FDM	Fused Deposition Modeling
FFF	Fused Filament Fabrication
LDPE	Low-Density Polyethylene
MEX	Material Extrusion
MFI	Melt Flow Index
NR	Natural Rubber
PA	Polyamide
PBAT	Poly(Butylene Adipate-co-Terephtalate)
PBS	Poly(Butylene Succinate)
PCL	Polycaprolactone
PET	Poly(Ethylene Terephthalate)
PETG	Glycol-Modified Poly(Ethylene Terephthalate)
PLA	Poly(Lactic Acid)
PS	Polystyrene
RA	Raster Angle
SEM	Scanning Electron Microscopy
TPU	Thermoplastic Polyurethane
